# Multidirectional Changes in Parameters Related to Sulfur Metabolism in Frog Tissues Exposed to Heavy Metal-Related Stress

**DOI:** 10.3390/biom10040574

**Published:** 2020-04-09

**Authors:** Marta Kaczor-Kamińska, Piotr Sura, Maria Wróbel

**Affiliations:** 1 Jagiellonian University Medical College, Faculty of Medicine, Chair of Medical Biochemistry, 7 Kopernika St., 31-034 Krakow, Poland; marta.b.kaczor@uj.edu.pl; 2 Jagiellonian University Medical College, Faculty of Health Sciences, Chair of Medical Biology, 7 Kopernika St., 31-034 Krakow, Poland; mbsura@cyf-kr.edu.pl

**Keywords:** antioxidative enzymes, lead, mercury, cadmium, oxidative stress, sulfurtransferases

## Abstract

The investigations showed changes of the cystathionine γ-lyase (CTH), 3-mercaptopyruvate sulfurtransferase (MPST) and rhodanese (TST) activity and gene expression in the brain, heart, liver, kidney, skeletal muscles and testes in frogs *Pelophylax ridibundus*, *Xenopus laevis* and *Xenopus tropicalis* in response to Pb^2+^, Hg^2+^ and Cd^2+^ stress. The results were analyzed jointly with changes in the expression of selected antioxidant enzymes (cytoplasmic and mitochondrial superoxide dismutase, glutathione peroxidase, catalase and thioredoxin reducatase) and with the level of malondialdehyde (a product of lipid peroxidation). The obtained results allowed for confirming the role of sulfurtransferases in the antioxidant protection of tissues exposed to heavy metal ions. Our results revealed different transcriptional responses of the investigated tissues to each of the examined heavy metals. The CTH, MPST and TST genes might be regarded as heavy metal stress-responsive. The CTH gene expression up-regulation was confirmed in the liver (Pb^2+^, Hg^2+^, Cd^2+^) and skeletal muscle (Hg^2+^), MPST in the brain (Pb^2+^, Hg^2+^), kidney (Pb^2+^, Cd^2+^), skeletal muscle (Pb^2+^, Hg^2+^,Cd^2+^) and TST in the brain (Pb^2+^) and kidney (Pb^2+^, Hg^2+^, Cd^2+^). Lead, mercury and cadmium toxicity was demonstrated to affect the glutathione (GSH) and cysteine levels, the concentration ratio of reduced to oxidized glutathione ([GSH]/[GSSG]) and the level of sulfane sulfur-containing compounds, which in case of enhanced reactive oxygen species generation can reveal their antioxidative properties. The present report is the first to widely describe the role of the sulfane sulfur/H_2_S generating enzymes and the cysteine/glutathione system in Pb^2+^, Hg^2+^ and Cd^2+^ stress in various frog tissues, and to explore the mechanisms mediating heavy metal-related stress.

## 1. Introduction

**Figure 1:**
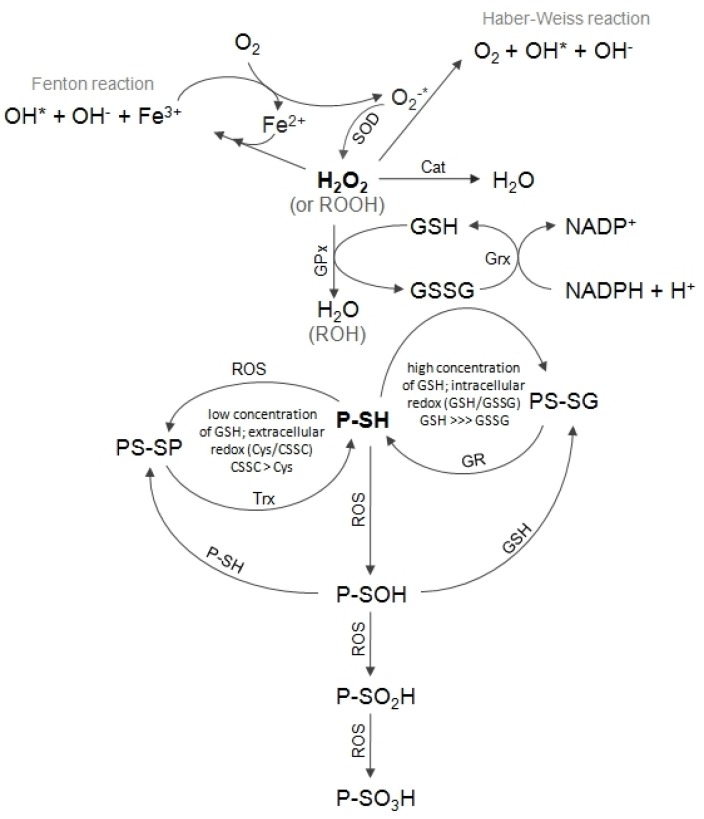
The thiol-based mechanism of antioxidant defense system (catalase (CAT); glutathione peroxidase (GPx); glutathione reductase (GR); thiol containing proteins (P-SH); reactive oxygen species (ROS); superoxide dismutase (SOD); thioredoxin (Trx)).

The biological effects of heavy metals are not completely clear, but there are several lines of evidence that oxidative stress is involved in perturbation of cell homeostasis and at high intensity leads to disturbances of cell function and, as a consequence, to tissue damage [1]. Cells are constantly exposed to low levels of free radicals and although their antioxidant defense mechanism (Figure 1) is able to handle them, all ionic heavy metals significantly contribute to the enhanced production of new ones. Redox-active metals such as Cu catalyze Haber–Weiss/Fenton reaction generating oxidizing radicals (Figure 1). The cellular targets for heavy metal toxicity especially include such organs as the kidney, liver, heart and testicles, as well as the immune and nervous systems [2,3,4,5].

Cells, tissues, organs and organisms utilize multiple layers of antioxidant defenses and damage removal, as well as replacement or repair systems in order to cope with the stress and damage that oxygen engenders (Figure 1). However, cellular antioxidant defense mechanisms could be insufficient and oxidative stress may occur. Oxidative stress may be a result of an increased exposition to oxidants or a decreased protection against oxidants, or even both problems occurring simultaneously [6].

Heavy metal-induced oxidative stress leads to a different type of damages. Nuclear damages caused by metal binding and redox reactions affect DNA repair and gene expression regulatory proteins [7]. Proteins are primary targets of heavy metal ions. Metals such as cadmium, lead and mercury, as well as redox reactions caused by them, are likely to exhaust the cellular antioxidant defense system and lead to the inhibition of major antioxidant enzymes (i.e., superoxide dismutase (SOD), catalase (Cat) and glutathione peroxidase (GPx)) or a decrease of low molecular weight antioxidants level, such as glutathione, ascorbate or tocopherol (Figure 1). Apart from the loss of ions and proteins, cadmium, lead and mercury efficiently inhibit the refolding of chemically denatured proteins. In the presence of the above metals, proteins misfold and aggregate. Loss of total protein content triggers biochemical changes in the cells [8]. Particularly susceptible to oxidative damages are the cell membranes containing unsaturated lipids. They are caused by lipid peroxidation that leads to hardening of lipids constituting the cell membrane. The damaged cell membrane changes the uptake of nutrients, the cell signaling system and many other cellular functions. The number of damaged cells directly affects the general function of tissues or organs.

Apart from reactive oxygen species (ROS) generation, there are three other main categories of heavy metal toxicity: 1) blocking the important functional groups in biomolecules by replacement of essential cations from specific binding sites in biomolecules [9], 2) direct interactions with enzymes or proteins due to their high affinities for thiol-, histydyl-, carboxyl- groups [9,10] and 3) changing the active conformation of biomolecules [10]. Enzymes that have metals on or close to their active sites are especially sensitive to metal-catalyzed oxidation. Mechanisms guarding cellular homeostasis could be broken by heavy metals ions binding to protein sites. In a protein, cysteine residues are more easily accessible to toxic agents due to the fact that the sulfur atom in the thiol group (-SH) of cysteine is the strongest nucleofile [11]. Therefore, thiols (RSH), including the proteins containing -SH groups, and non-protein low molecular weight compounds containing -SH groups undergo much faster oxidation in comparison with compounds containing hydroxyl- or amine- groups. A number of possible thiol derivatives can be produced endogenously, some of them being most well studied: S-nitrosation (-SNO), S-glutathionylation (-SSG) and S-sulfenylation (-SOH). Oxidation of sulfhydryl groups or methionine residues of protein causes conformational changes, protein unfolding and degradation [12]. In some cases, specific oxidation of proteins may take place (Figure 1). For example, methionine may be oxidized to methionine sulfoxide [13], phenylalanine to o-tyrosine [14]; sulfhydryl groups may be oxidized to form disulfide bonds [15] and metal-catalyzed oxidation may cause the formation of carbonyl groups that may be introduced into side chains of proteins [16]. Every single oxidative modification of enzymes changes or inhibits their activities; therefore, efficiently functioning cellular antioxidant defense systems are important. Hence, the cysteine residues of many enzymes cycle from a thiol to a disulfide are generated as a part of their catalytic cycles. The formation of disulfide protects them against irreversible oxidation, as S-S bonds can be reduced by thioredoxin (Trx)/thioredoxin reductase (TrxR, EC 1.8.1.9) system or glutathione (GSH)/glutharedoxin/glutathione reductase (Grx, EC 1.8.1.7) system (Figure 1) [17].

**Figure 2:**
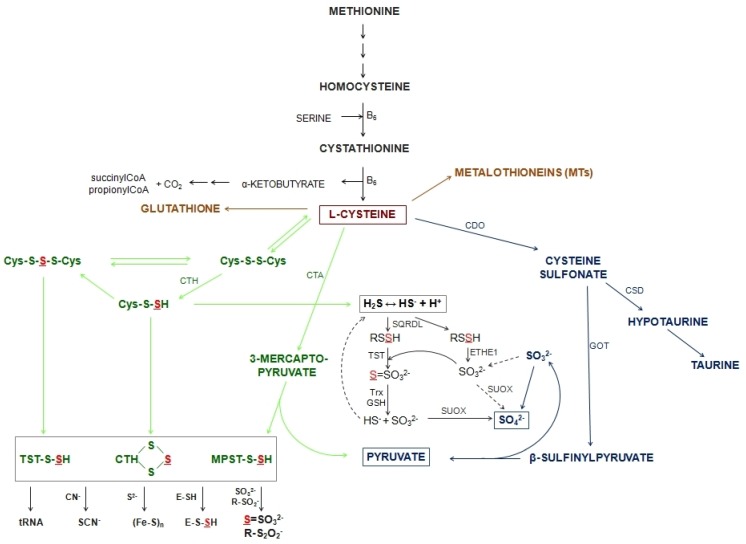
L-cysteine metabolism. Green color—the non-oxidative pathway of L-cysteine (Cys-S-S-S-Cys-thiocystine; Cys-S-S-Cys-cystine; Cys-S-SH-thiocysteine), blue color—the L-cysteine dioxygenase-initiated oxygen transformation pathway, gray color—the thiosulfate cycle that can produce hydrogen sulfide, brown color—other biologically important compounds for which the cysteine serves as a precursor, sulfane sulfur is marked in red (modified according to [19]). Proteins abbreviations: cystathionine dioxygenase (CDO), cysteinesulfinate decarboxylase (CSD); cystathionine γ-lyase (CTH); persulfide dioxygenase (ETHE1); aspartate aminotransferase (GOT); sulfide quinone reductase-like protein (SQRDL); sulfite oxidase (SUOX); thioredoxin (Trx); rhodanese (TST).

Cysteine (Cys) is the biochemical precursor of low molecular persulfides and H_2_S. Methionine is the sole essential source of sulfur in mammalian systems and can serve as a precursor for the synthesis of Cys and all other sulfur-containing molecules. The transsulfuration pathway is essentially the sole pathway of methionine catabolism under physiological conditions. Transsulfuration results in the transfer of sulfur from methionine to serine to synthesize Cys (Figure 2). Homocysteine and cystathionine are intermediates of the process. The transsulfuration of homocysteine to Cys is catalyzed by two pyridoxal 5’-phosphate (PLP)-dependent enzymes: cystathionine β-synthase (CBS, EC 4.2.1.22) and cystathionine γ-lyase (CTH, EC 4.4.1.1). CBS catalyzes the condensation of homocysteine and serine to form cystathionine in an irreversible reaction. Then, cystathionine is hydrolyzed by CTH and forms Cys, α-ketobutyrate and ammonia. Cys provides sulfur for the synthesis of key sulfur-containing molecules that function as important enzyme cofactors, GSH, metallothioneins (MTs) and iron-sulfur clusters in enzyme active-sites (Figure 2). The specific metabolic function of GSH should be highlighted—GSH serves as a reservoir of Cys and for Cys transport to extrahepatic tissues [18]. 

However, there are several pathways for Cys catabolism (Figure 2). Cys can be oxidized to cysteinesulfinate by cysteine dioxygenase (CDO, EC 1.13.11.20), which is decarboxylated to hypotaurine by cysteinesulfinate decarboxylase (CSD, EC 4.1.1.29). Hypotaurine is subsequently oxidized to taurine and CO_2_. Alternatively, cysteinesulfinate may be transaminated by aspartate aminotransferase (GOT, EC 1.8.3.1) and form β-sulfinylpyruvate, which gives rise to pyruvate and sulfite, which is readily oxidized to sulfate (Figure 2). These oxidation pathways lead to both taurine and sulfate formation, with sulfate being the dominant product [18]. Sulfite resulting from transaminative process is readily oxidized by sulfite oxidase (SUOX, EC 1.8.3.1) to sulfate. Sulfate can either be excreted in urine or be activated to 3’-phosphoadenosine-5’-phosphosulfate, which can serve as a sulfane donor for sulfate ester formation [18].

**Figure 3:**
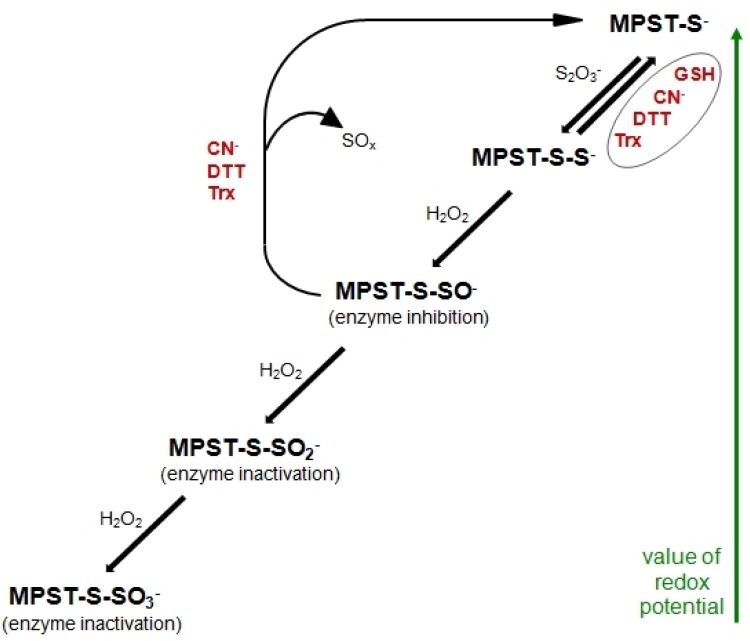
The redox cycle of the catalytically active Cys 247 residue of MPST (reduced glutathione (GSH); dithiothreitol (DTT); thioredoxin reductase (Trx); sulfur oxide (SO_x_)) (modified according to [22]).

Non-oxidative catabolism of Cys includes three different paths: 1) CTH can cause β-cleavage of Cys to yield pyruvate, ammonia and thiocysteine—thiocysteine can undergo further reactions, leading to sulfide release, 2) Cys can also be transaminated by aminotransferases to yield 3-mercaptopyruvate, which is further metabolized by mercaptopyruvate sulfurtransferase (MPST, EC 2.8.1.2) to release or transport the sulfur atom or 3) the thiol group of Cys can be substituted by diverse thiol compounds and form a corresponding thioether in a reaction catalyzed by CBS [18]. H_2_S may be a product of each of these desulfuration pathways and this might be an easy way of rapid removal of excess Cys from the body. H_2_S formed by Cys desulfhydration is further oxidized in the mitochondria. Sulfide oxidation is initiated by the action of mitochondrial sulfide quinone reductase-like protein (SQRDL), which forms an enzyme persulfide that is subsequently converted to sulfite by persulfide dioxygenase (ETHE1, EC 1.13.11.18) or transferred to sulfite by rhodanese (TST, EC 2.8.1.1) to form thiosulfate [20] (Figure 2). In the next step, thiosulfate is cleaved by the GSH-dependent thiosulfate reductase (TR, EC 2.8.1.3) activity to yield sulfite and release H_2_S [18]. Three sulfurtransferases, namely CTH, MPST and TST, participate in these pathways. All of them transfer reduced sulfur atoms from various donors (sulfane sulfur-containing compounds) to various acceptors. The reduced sulfur may be used in the synthesis of molecules requiring a source of reduced atoms or it may be oxidized to thiosulfate (inner sulfate atom), sulfite and finally sulfate. All three aforementioned enzymes have sulfhydryl groups in their active sites that can be blocked by heavy metal ions. Oxidation of these groups may result in inhibition of the activity of enzymes with redox-active Cys in the active site, but reduction with Trx or GSH can recover the activity of these enzymes (Figure 1, Figure 3) [21].

In our previous studies [4,5,23,24] we postulated that sulfurtransferases (CTH, MPST and TST) can exhibit antioxidative properties. In general, the obtained results remained in line with those presented previously; however, for the first time, in the present paper, we have shown changes of the sulfurtransferase activity results in comparison to the level of their gene expression. For the first time, these results were also combined with the results on changes in the expression of selected antioxidant enzymes and with lipid peroxidation level. These selected measurements allowed for a better understanding of the effect of lead, mercury and cadmium ions on non-oxidative sulfur metabolism and confirming the role of sulfurtransferases in the antioxidant protection of the organism exposed to their action.

## 2. Materials and Methods

### 2.1. Chemicals

The chemicals used in the experiments were purchased from Sigma-Aldrich (Chemical Company, St Louis, MO, USA). These were: agarose, albumin (from bovine serum, fraction V), mercuric chloride, chloroform, DL-cystathionine, piridoxal-5-phosphate, β-nicotinamide adenine dinucleotide reduced disodium salt hydrate, lactate dehydrogenase, dithiothreitol, N-ethylmaleimide, sodium chloride, sodium sulfite, sodium carbonate, sodium thiosulfate, L-glutathione reduced (GSH), glutathione oxidized form (GSSG), L-cysteine (Cys), L-cystine (CSSC), bathophenantrolinedisulfonic acid (BPDS), 2,4-dinitrofluorobenzene (DNFB), trifluoroacetic acid, acetonitrile, iron (III) nitrate nonhydrate, Ribonuclease Inhibitor from human placenta, isopropanol, Folin-Ciocalteu’s Phenol Reagent, formaldehyde and the Lipid Peroxidation (MDA) Assay Kit. The remaining chemicals, i.e., sodium dihydrogen phosphate dehydrate, potassium hydroxide, sodium hydroxide, ammonia solution, lead nitrate, potassium sodium tartrate tetrahydrate, disodium ethylenediaminetetraacetate dihydrate (EDTA), cadmium chloride 2.5-hydrate, copper (II) sulfate pentahydrate, ethanol and 70% perchloric acid were obtained from POCh S.A. (Gliwice), sodium 3-mercaptopyruvate from FLUKA AG, potassium cyanide was obtained from Merck (Darmstadt, Germany) and Nε-methyllysine from Bachem (Bubendorf, Switzerland). From Chempur (Piekary Śląskie, Poland), we obtained butanol, sodium dihydrogen phosphate dihydrate, ethanol, nitric acid, perchloric acid (PCA) and potassium bicarbonate; glacial acetic acid was delivered by PPH Stan lab (Lublin, Poland). Reagents dedicated to performing molecular studies were obtained from four different companies: 6 × DNA Loading Dye Buffer, DNTP Mix, Oligo(dT)_18_ Primer, 100 bp DNA Ladder and DEPC-treated Water from Fermentas (Canada), M-MuLV RT buffer 5×, Reverse Transcriptase M-MuLV from Roche (German), Nuclease-Free Water from Promega (USA) and 10× Dram Taq Buffer and Dram Taq DNA Polymerase from Thermo Scientific (USA).

### 2.2. Animals

To perform the study, three species of anuran amphibians were selected: *Pelophylax ridibundus*, *Xenopus laevis* and *Xenopus tropicalis*. Two of them: *Pelophylax ridibundus* (*Rana ridibunda*—the older name) and *Xenopus laevis* due to our earlier investigations [4,5,23,24] and the third one—*Xenopus tropicalis*—due to the fact that in the NCBI database its genome appeared first. The consent to acquire marsh frogs *Pelophylax ridibundus* was issued by the General Director of Environment Protection (DOPozgiz-4200 / II-77/3703/10 / JRO). The licenses were obtained from the Local Ethics Commission (43/OP/2005) and the Polish Ministry of Environment to perform studies on a protected species (ref. No: DOPogiz-4200/II-06/5453/05/aj). Both *Xenopus laevis* and *Xenopus tropicalis* were obtained from the commercial dealer. *Pelophylax ridibundus* individuals were collected in the vicinity of Krakow (southern Poland). We collected frogs of both sexes that were sexually matured and had maximal sizes, as only such individuals had tissues of enough size (especially testes, brain and mesonephros [kidneys]). They were placed in plastic aquaria for 1 week with dechlorinated tap water. The animals were kept at room temperature with a natural day/night rhythm. After acclimatization, the frogs were divided into four groups. Frogs in the control group were kept in clear water for following 10 days. The experimental groups were exposed to, respectively: nitrate lead (Pb(NO_3_)_2_) with a constant salt concentration of 28 mg/L (LC_50_ for frogs of the *P. ridibundus* species was determined at the level of 138 mg/L [25]), mercuric chloride (HgCl_2_) with a constant salt concentration of 1.353 mg/L (in the scientific literature, there is only one available piece of information concerning the value of LC_50_ after exposition of *X. leavis* embryos to mercury chloride and it is equal to 0.163 mg/L [26]) and cadmium chloride, 2.5-hydrate (CdCl_2_ 2.5 H_2_O) with a constant salt concentration of 40 mg/L (LC_50_ for frogs of the *P. ridibundus* species was determined at the level of 51.2 mg/L [27]). The used exposition concentrations are far above environmentally relevant concentrations. The frogs absorbed heavy metal ions from the contaminated water through their highly permeable skin. Stability of the media (water and dissolved metal salts) was provided by everyday changing of these exposure media on fresh ones. After 10 days, appropriate tissues were collected in keeping with the procedures approved by the Local Bioethics Commission in Krakow (Poland).

### 2.3. Experiments Design

Investigation using *Pelophylax ridibundus* were conducted to determine: 1) the content of lead, mercury and cadmium in various tissues (total individuals number—16, four experimental groups containing the following amount of individuals: the control group—five; the group exposed to lead-stress—three; mercury-stress—three and cadmium-stress—five; the number of individuals in group depends on the size of frogs), 2) the malondialdehyde (MDA) level in various tissues (total amount of individuals—15; the control group—four; the group exposed to lead-stress—four; mercury-stress—four and cadmium-stress—three), 3) the level of sulfane sulfur and the sulfurtransferases activity, as well as the level of low-molecular thiols using RP-HPLC methods (total individuals number—22). To perform the experiment, we had to acquire frog individuals twice in an interval of a few months because the entire population of frogs needed for the experiment was not successfully obtained at the first time. During the first acquisition of frogs, we collected 10 individuals that were divided in two experimental groups: the control group consists of four individuals and the group exposed to cadmium-stress consists of six individuals. The second time, we collected 12 individuals of *Pelophylax ridibundus* and divided them in three experimental groups: the control group and the groups exposed to lead- and mercury-stress. Each group consisted of four frogs. Unfortunately, since the *Pelophylax ridibundus* individuals caught during the second expedition were smaller, we were not able to complete the determination of low-molecular thiols by RP-HPLC method using them. According to the consent to acquire *Pelophylax ridibundus* frogs, we had restrictions concerning the size of the population that we could catch. Therefore, in the RP-HPLC measurements, both missing groups were completed by seven *Xenopus tropicalis* frogs (the control group—three individuals and the mercury-stress group—four individuals), which are commercially available. We decided to use this frog species since *Xenopus tropicalis* individuals were included by us earlier in the experiment of selected genes expression detection. In the genes’ expression experiment, we used 19 frogs divided in four experimental groups: the control group—four individuals, the group exposed to lead-stress—five; mercury-stress—five and cadmium-stress—five. The tissues of the third species of frogs—*Xenopus leavis* (six individuals)—were only used to make the calibration curves for the RP-HPLC. Both *Xenopus* species are fully aquatic, and easy to maintain in the laboratory (if necessary). The experiments carried out on the tissues of these animals help us to avoid individual differences, as is in the case in animals obtained in their natural environment.

### 2.4. Tissues Collection

After 10 days, the frogs were decapitated and their spinal cord was pitched. The procedure was approved by the Local Bioethics Commission for the experiments on animals in Krakow, Poland (Resolution No. 92/2009). For further studies, we excised the brain, liver, heart, kidney, testes and muscle from the thigh. The tissues were washed out in cold saline, immediately frozen in liquid nitrogen and kept at a temperature of −80 °C until assays. Before assays, the tissues were homogenized (Ultra-Turrax T 25; Janke & Kunkel IKA-Labortechnik Company, Staufen, Germany) separately in four volumes of appropriate solution (0.1 M phosphate buffer pH 7.5; 1 mM BPDS/10% PCA or TRIZOL) and centrifuged. The supernatant was used for the determination of enzyme activities and sulfane sulfur level, determination of low molecular weight sulfur-containing compounds using RP-HPLC and studies of gene expression.

### 2.5. Quantity Determination of Heavy Metal Ions Content in Particular Frog Tissues

To perform a quantitative determination of heavy metal ions content in frog tissues, the tissue preparation procedure was adapted to the needs of the assay. In the first step, the tissues were lyophilized. Then, 7 ml of 65% nitric acid and 1 ml of 30% hydrogen peroxide were added. Samples were extracted using the microwave digestion system (Milestone Microwave Laboratory Systems, Digestion, Application Note DG-CL-02) at 200 °C, according to the following mineralization program: 1) 200 °C, 10 min, power = 1000 watts, 2) 200 °C, 20 min, power = 1000 watts. After completion of extraction, the samples were transferred from teflon vessels to measuring flasks using distilled water. Each flask was filled up with distilled water to the final volume of 20 ml. 

The values of the cadmium and mercury ions content were determined by the atomic absorption spectrometry (Unicam ICE 3500). To quantitatively determine both these metals, two different atomizers: a flame atomizer (cadmium; F-AAS) and a hydride atomizer (mercury; Hg-AAS) were required. This part of the research was conducted in cooperation with the Department of Environmental Protection, Faculty of Geology, Geophysics and Environmental Protection, AGH University of Science and Technology in Krakow, Poland.

Lead ions content in particular frogs’ tissues was determined using the ICP-MS (inductively coupled plasma mass spectrometry) technique. The study was conducted in cooperation with the Laboratory of Industrial Measurements and Environment, Institute of Ceramics and Building Materials, in Opole, Poland.

### 2.6. Quantity Determination of Low Molecular Sulfur-containing Compounds, Malondialdehyde (MDA) and Biochemical Activity Assay

The MPST activity was assayed according to the method of Valentine and Frankenfeld [28] with some modifications described by Wróbel et al. [29]. The TST activity was assayed by the Sörbo method [30]. The assays were carried out according to the procedure described by Wróbel et al. [29]. The CTH activity was determined according to Matsuo and Greenberg [31] with the modification described by Czubak et al. [32]. Sulfane sulfur was determined by the method of Wood [33], based on cold cyanosis and colorimetric detection of ferric thiocyanate complex ion. Protein content was determined by the method of Lowry et al. [34] using crystalline bovine serum albumin as a standard. The RP-HPLC method of Dominic et al. [35] with the modification described by Wróbel et al. [36] was used to determine the level of reduced (GSH) and oxidized form (GSSG) of glutathione, Cys and cystine (CSSC). Standard curves were generated in the supernatant obtained from tissue homogenates in the range from 13 to 75 nmol of each compound per mL. Determination of the malondialdehyde (MDA) content in the tissues was carried out according to the Sigma-Aldrich protocol, using the Lipid Peroxidation (MDA) Assay Kit.

### 2.7. Isolation of Total RNA

Total RNA was extracted from the tissues using TRIZOL, according to the protocol provided by the manufacturer. The extracted RNA was suspended in ribonuclease free-water and quantified by measuring the absorbance at 260 nm. After the isolation procedure, every time, the purity of the obtained RNA was checked. This parameter was determined as the ratio of the absorbance: A_260_ nm/A_280_ nm. Until further studies were performed, RNA solutions were stored at −80 °C.

### 2.8. Reverse Transcription of RNA

Total RNA from particular tissues was reverse-transcribed using the Aid^TM^ H Minus First Strand cDNA Synthesis Kit according to the instructions provided by the manufacturer. For reverse transcription (RT), 2 μg of total RNA was mixed with 1 μL Oligo d(T) primer (0.5 μg/μL) and water pretreated with diethylpyrocarbonate (DEPC-H_2_O) and incubated for 5 min at 70 °C. After preincubation, other components were added to the mixture: 4 μL 5 × concentrated RT buffer (250 mM Tris-HCl, 250 mM KCl, 20 mM MgCl_2_, 50 mM DTT, pH 8.3 at 25 °C), 2 μL deoxyribonucleotide triphosphates (dNTPs, 10 mM) and 1 μL RNase inhibitor (20 U/μL). After incubation at 37 °C for 5 min, 1 μL RevertAid M-MuLV Reverse Transcriptase (200 U/μL) in a total volume of 20 μL was added. The mixture was first incubated for 60 min at 42 °C, then for the final 10 min at 70 °C. If necessary, the solutions of complementary DNA (cDNA) were stored at −20 °C.

### 2.9. Polymerase Chain Reaction (PCR)

The expression of nine different genes (CTH, MPST, TST, GAPDH—glyceraldehyde 3-phosphate dehydrogenase—cytoplasmic SOD, mitochondrial SOD, GPx, Cat, TrxR) was analyzed by PCR. Amplification of cDNA was run in a 25 μL reaction volume that contained the following: 2 μL of synthesized cDNA, 10 μM of each of gene-specific primer pair (Table 1), 2 U/μL DNA polymerase in 10 mM buffer Tris-HCl, pH 8.8 (supplemented with 1.5 mM MgCl_2_, 50 mM KCl, 0.1% Triton X-100), 10 mM of each dNTPs and DEPC-H_2_O. 

PCR conditions for particular target genes are shown in Table 2. In each case, a similar reaction was also performed in the mixture without DNA (negative control) in order to confirm the specificity of the obtained reaction products. All amplification reactions were performed at least three times to ensure the reproducibility of results. As a reference gene GAPDH was used. All PCR products were analyzed by electrophoresis on a 2.5% agarose gel stained with ethidium bromide and directly visualized under UV light and photographed. 

### 2.10. Statistical Analysis

The statistical significance of differences between the experimental group and the controls were determined using the Student’s t-test. The differences were regarded as significant at *p* < 0.05.

## 3. Results

### 3.1. Heavy Metal Accumulation in Selected Pelophylax Ridibundus Tissues

Using high-throughput methods of analysis, we confirmed that heavy metal ions showed a tendency to accumulate in frog tissues (Table 3). Studies performed using the ICP-MS for lead, Hg-AAS for mercury and F-AAS methods for cadmium demonstrated that all the analyzed tissues accumulated heavy metal ions after 10-day exposition. The analyses were performed in the brain, heart, liver, kidneys, skeletal muscle and testes and showed that the accumulation occurred in all these tissues. In all the cases, the highest accumulation of heavy metal ions occurred in the kidneys and liver. In the kidney, the contents of heavy metal ions after the 10-day exposition was increased 17 times for the lead ions, while mercury ions caused a 2820-fold and cadmium ions–a thirty-one-fold increase as compared to values obtained for the control group. After 10-day exposition, liver accumulation of heavy metal ions increased as compared to the control group in the following manner: lead—18 times; mercury—373 times and cadmium—approximately 2.9 times. A relatively large amount of heavy metal ions was also accumulated in the heart, testes and brain. In all the examined tissues in the control group, cadmium was present; furthermore, the liver and kidneys additionally contained lead and mercury, which proves that the animals were exposed to heavy metal ions in their natural living environment.

### 3.2. Sulfane Sulfur Levels in Selected Tissues

In all the investigated tissues of controls the determined levels of sulfane sulfur were similar, being in the range of 136–192 nmol/mg protein (Table 4), except the skeletal muscle, where the level was ca. half of these values. After 10 days of exposition to heavy metal ions in the heart, testes (lead) and skeletal muscle (lead and cadmium), the sulfane sulfur level was comparable with the level determined in the control group (Table 4, graphical presentation of these results are also included in the Supporting information file—Figure S1). The said level was significantly increased in the kidney (lead and cadmium) (Table 4, Figure S1 Supporting information), as well as in the testes and skeletal muscle of frogs exposed to mercury ions. In contrast, the sulfane sulfur level was significantly decreased in the kidney after exposition to mercury ions, in the testes exposed to cadmium ions and in all these cases in the liver (Table 4, Figure S1 Supporting information). The obtained results were closely related to Cys and GSH contents determined in particular tissues during our studies (Figure S1 Supporting information).

### 3.3. Changes in Activity and Expression of Three Sulfurtransferases in Response to Lead, Mercury and Cadmium Exposition

#### 3.3.1. Rhodanese (TST)

The TST activity in particular tissues of *Pelophylax ridibundus* exposed to heavy metal ions for 10 days are presented in Table 4 (graphical presentation of these results is also included in the Supporting information file—Figure S2). Based on these results obtained in the control groups, we can observe that the highest activity of TST was observed in the kidney and liver, while the lowest was observed in the testes (Table 4). The exposition to mercury ions (in the investigated concentrations and experimental time) did not affect the TST activity, which was maintained at the control level in all the tissues except the liver, in which the activity increased (Table 4, Figure S2 Supporting information). The TST expression (detected as changes in the mRNA level) in the investigated tissues of *Xenopus tropicalis* is presented in Table 5 and Figure S2 in the Supporting information file. In the kidney, the TST expression was increased (in comparison to the controls), but the activity of the enzyme remained unchanged (Table 4, Figure S2 Supporting information). In the heart and skeletal muscle exposed to cadmium ions, the TST activity decreased (Table 4, Figure S2 Supporting information), what was correlated with the decreased expression in the heart (Table 5). The TST expression was low in the testes and comparable with the control group when the tissue was exposed to cadmium ions (Table 5, Figure S2 Supporting information), while its activity was higher as compared to the controls (Table 4, Figure S2 Supporting information). In the heart exposed to lead ions (Table 4, Figure S2 Supporting information), a significant increase in the TST activity was found (*p* < 0.001), while the activity in the skeletal muscles decreased. Simultaneously, the TST expression in the skeletal muscles remained unchanged as compared to the control group, whereas an increase in TST expression was noted in the kidney and brain (Table 5, Figure S2 Supporting information).

#### 3.3.2. Mercaptopyrvate Sulfurtransferase (MPST)

As far as changes in the MPST activity in particular tissues of *Pelophylax ridibundus* are concerned, it was observed that exposition to cadmium and lead ions changed the MPST activity (Table 4). Graphical presentation of these results is also included in the Supporting information file—Figure S3. The MPST activity significantly (*p* < 0.01) decreased in the heart, kidney, skeletal muscle response to both ions, in the liver in response to cadmium ions and in the testes in response to lead ions (Table 4, Figure S3 Supporting information). The decrease of the MPST activity was also observed in the brain and heart exposed to mercury ions (Table 4, Figure S3 Supporting information). Mercury ions caused the greatest decrease in the activity of the enzyme—the MPST activity dropped to a half of the value determined for the control group after 10 days of exposition to this metal ions (Table 4, Figure S3 Supporting information). In the other cases, the obtained values of the MPST activity remained at the control level. In only one case was a parallel decrease of the MPST expression (mRNA levels) noted—in the heart exposed to cadmium ions for 10 days (Table 4 and Table 5, Figure S3 Supporting information). In the testes exposed to mercury ions, the MPST expression level was also decreased (Table 5, Figure S3 Supporting information), but the MPST activity remained unchanged (Table 4, Figure S3 Supporting information). In comparison with the control group, the increased MPST expression was also detected in the brain exposed to lead and mercury ions, in the kidney exposed to lead and cadmium ions, as well as in the skeletal muscle in case of all the three ions (Table 4, Figure S3 Supporting information). Despite the MPST gene expression growth in the kidney and skeletal muscle exposed to lead and cadmium ions, as well as in the brain exposed to mercury ions (Table 5, Figure S3 Supporting information), we observed a decrease in the enzyme activity (Table 4, Figure S3 Supporting information).

#### 3.3.3. Cystathionine γ-Lyase (CTH)

The results obtained from the CTH activity determination in *Pelophylax ridibundus* tissues are shown in Table 4 (a graphical presentation of these results is also included in the Supporting information file—Figure S4). Based on the data, it can be observed that the CTH activity was increased in the brain exposed to all the examined heavy metal ions; it was also increased in the liver exposed to lead and mercury ions, as well as in the skeletal muscle and testes exposed to mercury ions after 10 days of exposition (Table 4, Figure S4 Supporting information). In the heart exposed to mercury ions and in the kidney exposed to cadmium ions—the activity of CTH decreased in comparison to the value determined in the control group (Table 4, Figure S4 Supporting information). These changes in the CTH activity were statistically significant (*p* < 0.01). As far as the CTH expression was concerned (Table 5, Figure S4 Supporting information), it cannot be that in the brain, testes and liver exposed to lead and cadmium ions, the CTH expression was higher in comparison with the expression in the control group. Moreover, in the liver, the CTH expression was higher in the experimental group exposed to mercury ions. Ten days of exposition to mercury ions led to the increased CTH expression in the heart and skeletal muscle (Table 5, Figure S4 Supporting information). In only one case was the decreased CTH expression observed—in the skeletal muscle exposed to lead ions (Table 5, Figure S4 Supporting information). This decrease of the CTH expression did not affect changes in the activity of CTH enzyme (Table 4, Figure S4 Supporting information). 

#### 3.3.4. Antioxidative Enzymes

Another objective of the study was the investigation of gene expression of some enzymes involved in defense against oxidative stress—cytoplasmic and mitochondrial SOD, GPx, Cat and TrxR. The obtained results are presented in Table 5. The expression (mRNA levels) of some enzymes increased what can suggest a response to an elevation of ROS in tissues of animals exposed to the presence of heavy metals in water. The exposition to mercury ions led to an increase in the GPx expression in the brain, kidney and skeletal muscle (Table 5). Especially in the kidney, the GPx expression level was remarkably increased after 10 days of exposition to mercury ions (Table 5). In the testes only did the exposition to mercury ions result in a decrease in the GPx expression (Table 5). The expression of TrxR was also higher in the kidney (cadmium ions), skeletal muscle (mercury ions) and liver (lead, mercury and cadmium ions) as compared to the controls (Table 5). On the other hand, in the testes, cadmium and mercury ions caused a decrease of the TrxR expression in comparison to the control group (Table 5). A similar effect could be observed in the brain; additionally, in the cerebral tissue, the decrease was caused by lead ions. In the heart and liver, an increase of the Cat expression was observed after exposition to mercury ions. In the kidney, an increase of the expression was caused by lead and cadmium ions, while the same heavy metals caused a decrease of the Cat expression in the brain. All three metal ions resulted in decreasing the Cat expression in the testes (Table 5). A decrease in these antioxidant enzymes expression in response to heavy metals exposition can suggest changes in their activity. The lowest changes were observed in the expression of the mitochondrial and cytoplasmic form of SOD genes. In the kidney only did the expression of mitochondrial SOD decrease in response to mercury ions (Table 5), while the expression of the cytoplasmic form decreased in the heart in presence of cadmium ions and increased in the skeletal muscle in presence of mercury and cadmium ions (Table 5).

### 3.4. GSH and Cys Content in Selected Tissues

Our results obtained from HPLC measurements (Table 6) showed that exposition to lead ions [28 mg/L, 10 days] exerted a weaker effect on the GSH and Cys levels in selected tissues than exposition to mercury [1.353 mg/L, 10 days] or cadmium ions [40 mg/L, 10 days]. The GSH and Cys levels were at the control level after 10 days of exposition to lead in the brain, heart, as well as kidney. In the skeletal muscles, a decrease in the Cys level was observed after 10 days of exposition to lead ions, whereas its level was increased (2.1 times) in the liver. At the same time, in these tissues, the level of GSH did not change under the experimental conditions. Taking into account the exposition to mercury ions, the results indicated that in the brain and testes, the GSH as well as Cys levels decreased after 10 days of exposition, by, respectively, 2.6 and 2.5 times for the brain and 1.3 and 1.2 times for the testes. The level of Cys was also somewhat reduced in the skeletal muscle (the GSH level remained unchanged) after exposition to mercury ions, in contrast to the kidney and heart, where the Cys level increased 1.03 and 1.07 times, respectively. In the liver and kidney of animals treated with mercury ions, an increase of the GSH level was observed (4.2- and 1.5-fold, respectively), while a slightly decreased level of GSH was detected in the heart. The obtained results (in the group exposed to cadmium ions) demonstrated that in the brain, liver and skeletal muscle, the GSH level remained unchanged (as well as the Cys level in the brain and kidney), while in the heart, kidney and testes its level were, respectively, 1.45, 4.85 and 4.37 times higher in comparison with the level determined in the control group. We also observed that the Cys level was two times higher in the liver and testes in comparison with the control group. In the skeletal muscle only, a diminished Cys level was observed in response to the adverse experimental conditions caused by cadmium ions. Irrespective of the determination of low molecular weight thiols concentration, the concentration ratio of reduced to oxidized glutathione [GSH]/[GSSG] was determined in all the investigated tissues. The value of this ratio was used as an indicator of the cellular redox state, because it determines the antioxidative capacity of cells. Our results indicated that the [GSH]/[GSSG] ratio was increased after 10 days of exposition to mercury ions in all the examined tissues, except the brain and testes, where the ratio was, respectively, lower (two times) and unchanged. The opposite was observed in almost all the tissues exposed to lead ions. It seems that the tested concentration of lead ions exerted no effect on the [GSH]/[GSSG] value, and only in the heart a slightly increased ratio was observed. In the case of cadmium ions, the value of [GSH]/[GSSG] ratio increased, respectively, 2.5, 1.8 and 2.6 times in the liver, kidney and testes, seemed to be slightly decreased in the heart and showed no effect in the brain.

### 3.5. MDA Level in Selected Tissues

One of the objectives of the study was checking whether lipid peroxidation occurred after 10 days of exposition to heavy metal ions. Therefore, the MDA content was determined in particular tissues at the end of exposition. The results in Table 7 show the content of MDA oscillating around the control values for all the tissues except the brain exposed to lead and mercury ions, and the liver exposed to cadmium ions, for which the detected MDA content was reduced, and the liver exposed to lead ions, for which the content of MDA was slightly increased. 

## 4. Discussion

Heavy metal compounds are major environmental pollutants that cause stress in living organisms. This is due to the fact that lead, mercury and cadmium and the compounds that contain them are not biodegradable. Bioaccumulation of toxic metals in the soils and plants poses a direct risk to animals and human health [37]. Our results confirm heavy metal ions accumulation in all the studied *Pelophylax ridibundus* tissues after 10 days of exposition (Table 3). The final level of heavy metal ions in particular tissues of animals depends on their uptake, which in turn is determined by the combined action of physical barriers (e.g., cell membranes), the activity of enzymes metabolizing these compounds, the level of metal-binding proteins (metallothioneins, MTs) and they affinity to particular divalent metal cations, as well as, rate of metal-MTs degradation [38]. The induction of MTs expression by heavy metals may result in their subsequent accumulation in the cell. Experiments carried out using the ICP-MS (lead), Hg-AAS (mercury) and F-AAS (cadmium) methods show that the largest amounts of the analyzed heavy metal ions were accumulated in the kidneys and then in the liver (Table 3). The liver and kidney are two major organs involved in detoxification and elimination of most xenobiotics. The fate of particular heavy metal compounds depends on some of their properties (e.g., pKa value, lipophilicity). Lipophilic compounds tend to accumulate in the adipose tissues and acidic forms of these compounds tend to accumulate in the muscle (the physiological pH value of the muscles is approximately 6.5 [39]). It could be supposed that certain amount of heavy metal ions in muscles of experimental animals was transformed into their acid derivatives. The accumulation of heavy metal ions was confirmed in the skeletal muscles after 10 days of exposition (Table 3). The accumulation of mercury and cadmium also takes place in the brain and testes Table 3. Accumulation in the testes is higher than in the brain, however, the content of all the investigated ions in these tissues is much lower in comparison to their content in the kidney and/or liver. The results are in accordance with the earlier data obtained using the X-ray fluorescence spectroscopy (XRF) and Energy Dispersion Spectrum (EDS) methods [4,5].

Based on the results presented in Table 6, it can be stated that under the experimental conditions, the oxidative stress was confirmed in the heart, kidney, liver and skeletal muscles of animals exposed to mercury, and in the kidney, liver and testes of animals exposed to cadmium. Oxidative stress results in the increased levels of free radicals, peroxides and organic hydroperoxides, which, in turn, can result in an increased expression of enzymes involved in antioxidative defense mechanisms [40,41,42]. This is a reason why we investigated the expression of such enzymes as cytoplasmic SOD, mitochondrial SOD, Cat, GPx and TrxR. Our results seem to confirm ROS occurrence (Table 5). As is shown in Table 5, we observed changes in tissues expression of the investigated antioxidative enzymes. The expression of cytoplasmic SOD was higher in the skeletal muscles (mercury) as compared to the control group (Table 5). Interestingly, we found different effects of mercury ions exerted on the expression of cytoplasmic SOD and mitochondrial SOD in the kidney (Table 5). The cytoplasmic SOD was up-regulated in the tissue following mercury uptake, while the mitochondrial SOD was down-regulated (Table 5). This can be explained by distinct mechanisms of regulation for cytoplasmic and mitochondrial SOD [43]. Hydrogen peroxide (H_2_O_2_)_,_ a product of a SOD-catalyzed reaction (Figure 1), is rapidly removed to protect cells against its deleterious action. In cells this is accomplished by two enzymes: GPx and Cat (Figure 1). Increased levels of Cat, the result of gene transcriptional activation, were found in H_2_O_2_-stressed cells [44,45]. The results presented in Table 5 confirm the involvement of these antioxidative enzymes in detoxication processes—in comparison to the control group, the expression of Cat, GPx and TrxR genes was increased in the kidneys in response to exposition to cadmium, whereas the exposition to mercury resulted in an increase of the GPx expression. In the skeletal muscles, the GPx expression was also increased in response to mercury. Additionally, an increase in the TrxR expression was noted in the liver (mercury and cadmium), and Cat in the heart (mercury). The increased expression of both Cat and GPx may be regarded as a defense against the elevated intracellular levels of H_2_O_2_ (Figure 1). GPx requires the reducing power of GSH to detoxify hydrogen peroxide (Figure 1). ROS alter the ratio of [GSH]/[GSSG], which can change the activity of many proteins because of the formation of mixed disulfides between GSH and protein Cys residues (glutathionylation) [46]. The disruption of the cellular thiol redox homeostasis is a good indicator of oxidative stress in an organism. When the cell is exposed to sustained oxidative stress, the formation and accumulation of mixed disulfides between protein thiols may occur, which can be re-reduced by TrxR. TrxR catalyzes the NADPH-dependent reduction of the active disulfide site of Trx to a dithiol, and in this way, it participates in the regulation of the redox state of cells (Figure 1) [47]. The results presented in Table 5 indicate that the Trx-TrxR system is actively operating in tissues exposed to heavy metals stress. Possibly, the Trx-TrxR system takes part in the defense of tissues from external noxious stimuli, similar to heavy metal ions, which can induce the formation of ROS. The Trx-TrxR system takes part in neutralization of ROS together with SOD, Cat and GPx. In the testes only, the expression of selected antioxidant genes (GPx, Cat and TrxR) decreased (Table 5). These can result in the elevated levels of ROS, leading to oxidative damage in the testes, the cellular redox homeostasis disturbance and a higher vulnerability to oxidative stress.

There are several markers of tissue oxidative damage, including MDA (a marker of lipid peroxidation). The results presented in Table 7 indicates the highest MDA levels in the liver and kidney, the lowest—in the skeletal muscle, after 10-day long exposition to heavy metal ions. These results remain in accordance with the results obtained by Šuran et al. [48]. Due to the fact that lead, mercury and cadmium accumulate mostly in the liver and kidney (Table 3), it can be expected that the levels of MDA would be higher there then in the muscle, where the heavy metals accumulation is much lower. The highest MDA levels were measured in the liver which contains far more lipids than other tissues. Additionally, in the liver, we observed much lower levels of MDA in the group exposed to cadmium as compared to the controls (Table 7). We also observed a decrease of MDA levels in the brain (lead, mercury) (Table 7). The possible explanation lies in different concentrations of MTs in tissues.

When the redox state in cells is maintained, the ratio [GSH]/[GSSG] is high. GSH participation in the xenobiotics metabolism (Figure 1) involves 1) conjugating electrophilic metabolites [11]; 2) scavenging free radicals and 3) acting as cofactor in the metabolism of formaldehyde (produced through lipid peroxidation and the metabolism of xenobiotics) [49]. The metabolism of the electrophilic metabolites and/or ROS proceeds with a corresponding depletion of GSH. When the rate of consumption exceeds that of synthesis and/or reduction, the level of available GSH decreases and destructive processes are progressing. The results presented in Table 6 show that stress caused by mercury ions resulted in the depletion of both GSH and GSSG, as well as total glutathione content in the brain, heart and testes. Exposition to lead caused depletion of GSSG only in the brain and heart (Table 6). Additionally, in the heart, the total level of GSH was also decreased (lead) (Table 6). As the total level of glutathione and level of its reduced form decreases, a smaller amount of the substances is available to scavenge radicals that arise from normal aerobic intermediary metabolism, and peroxidative damage occurs. In the heart (lead, mercury) and skeletal muscle (mercury), the [GSH]/[GSSG] ratio increased (Table 6). This means that the tissue antioxidant capacity was running out and oxidative stress had occurred. In the brain (mercury), testes (mercury) and skeletal muscle (lead, mercury, cadmium), a decrease in the Cys level was also observed (Table 6). Therefore, we can suspect that in tissues exposed to lead and mercury ions, the GSH level was decreased because of two reasons: 1) boosting oxidizing processes efficiency in the cells by increased ROS generation; 2) limiting the availability of Cys, which is a rate-limiting precursor to new glutathione molecules synthesis. Contrary to mercury and lead effects, cadmium increased the levels of GSH and GSSG, as well as the total level of glutathione in the heart and testes (Table 6). Additionally, oxidative stress occurrence was noted in the testes, despite the fact that the level of Cys increased and Cys could be redirected to the GSH synthesis pathway (Table 6). On the other hand, in the two main detoxification organs, i.e., the liver and kidney, all three heavy metals (lead, mercury and cadmium) trigger similar defense mechanisms that try to eliminate their toxic effects. The liver response to lead- and cadmium-stress involves raising the Cys levels (Table 6), but the excess of available Cys can be used multi-directionally. In the liver, the sulfane sulfur level was decreased in all cases of heavy metal-stress (Table 4, Figure S1 Supporting information). Based on these results, it could be stated that this excess of Cys is used to maintain the GSH level that can provide cellular antioxidant defense. It seems that this mechanism works properly in the group exposed to lead ions because the levels of GSH and GSSG, as well as total glutathione remained unchanged, but in the cadmium-treated group the oxidative stress was noted (Table 6). The animal exposition to mercury-stress elicits a similar cellular response in the liver and kidneys: we observed in both cases increased levels of reduced and total glutathione (Table 6). Oxidative stress was found in these tissues after 10 days of exposition to mercury ions (Table 6). However, in the kidneys, these elevated levels of GSH can be explained by the increased Cys level that can be used in GSH synthesis, as well as in sulfane sulfur-containing compounds formation (the elevated level of sulfane sulfur, Table 4, Figure S1 Supporting information). On the other hand, in the liver these elevated levels of GSH seemed to be associated with the Cys level, even if its level remained unchanged (Table 6). Because the sulfane sulfur levels were statistically decreased in the liver in all the cases of exposition (Table 4, Figure S1 Supporting information), we can assume the Cys redirection to the synthesis of GSH. The same was observed in the kidney of animals exposed to cadmium (Table 4, Figure S1 Supporting information, Table 6). Summarizing the results, chronic exposition to heavy metals can modulate the glutathione-mediated pathways in different ways, depending on the heavy metal.

Heavy metals are toxic to biological organisms because they interfere with redox cycling, deplete GSH and form complexes with S, N, O atoms in proteins. The most important ligands are the thiol groups of Cys residues and the imidazole groups of histidine residues because they produce the most stable complexes [50]. For this reason, sulfur metabolism is profoundly affected by heavy metals. Adaptation of rates of sulfur metabolic pathways and their directions is crucial for survival of organisms under stress conditions. Based on the results collected in Table 4 and Figure S1 in the Supporting information, we can state that heavy metal-stress statistically caused a significant increase in the levels of sulfane sulfur in the brain (lead, mercury, cadmium), kidney (lead, cadmium), skeletal muscle (mercury) and testes (mercury). The opposite effect—a decrease of sulfane sulfur level—can be observed in the liver (lead, mercury, cadmium), kidney (mercury) and testes (cadmium) (Table 4, Figure S1 Supporting information). The increase of sulfane sulfur-containing compounds may suggest that Cys (Figure 2) is redirected to non-oxidative sulfur metabolic pathways, instead of GSH synthesis. In the brain (lead, mercury, cadmium), kidney (lead) and skeletal muscle (mercury), sulfane sulfur was elevated while the GSH level remained unchanged (Table 4, Figure S1 Supporting information, Table 6). Contrariwise, the decreasing sulfane sulfur level in the kidney (mercury), liver (mercury) and testes (cadmium) was related to the increasing GSH level (Table 4, Figure S1 Supporting information, Table 6). It may suggest slowing-down non-oxidative sulfur metabolic processes and redirecting of available Cys to the GSH synthesis pathway. 

The changes in sulfane sulfur levels are related to changes in the expression and/or activity of enzymes participating in its turnover (TST, MPST, CTH). TST is responsible for transferring sulfur atoms from various donors (sulfane sulfur-containing compounds) to various acceptors [51], while MPST catalyzes the transfer of sulfane sulfur atom from 3-mercaptopyruvate to various acceptors, producing sulfane sulfur-containing compounds (e.g., thiosulfate), or releases it as hydrogen sulfide [51,52]. On the other hand, CTH is involved in sulfane sulfur formation in the cells [53]. An elevated sulfane sulfur level seems to have an impact on the increase in the CTH activity in three cases: in the brain (lead, mercury, cadmium), skeletal muscle (mercury) and testes (mercury) (Table 4, Figure S4 Supporting information). The results collected in Table 5 demonstrated that heavy metal-stress up-regulated the expression of gene encoding CTH in the brain (lead, cadmium), liver (lead, mercury, cadmium), skeletal muscle (mercury) and testes (lead, cadmium). Up-regulation of the CTH expression may consist in the cell response to the necessity of intensifying CTH catalyzed processes (the protein demand) because of a greater sulfane sulfur-containing compound production. It can be expected that along with the increase in the sulfane sulfur level (and, as a consequence, sulfane sulfur-containing compounds), there will be an additional increase in the activity of enzyme involved in its formation. In the liver, we also noted an increase in the CTH activity (lead, mercury); however, this increase was not directly related to an increased production of sulfane sulfur-containing compounds, because the level of sulfane sulfur in the hepatic tissue was decreased in each of the three cases (Table 4, Figures S1 and S4 Supporting information). On the other hand, in the kidneys of animals exposed to cadmium ions, the CTH activity was decreased despite the fact that the sulfane sulfur level was elevated (Table 4, Figures S1 and S4 Supporting information). A similar situation (the decrease of CTH activity), with the exception of the elevated level of sulfane sulfur, can be observed in the heart exposed to mercury impact (Table 4, Figures S1 and S4 Supporting information). In these two cases, the inhibition of CTH activity is on the protein level and seems to be directly associated with cadmium and mercury influence. The decrease in its activity could be a result of bonding and blocking of -SH groups in their active sites by heavy metal ions or of their oxidation to -SOH (Figure 3) in the presence of an increased concentration of ROS (Table 5). Taking into consideration enzymes participating in sulfane sulfur turnover, we can note that heavy metal-stress decreased the activity of MPST in the following cases: in the brain (mercury), heart (lead, mercury, cadmium), kidney (lead, cadmium), liver (cadmium), skeletal muscle (lead, cadmium) and testes (cadmium) (Table 4, Figure S3 Supporting information). The cells try to cope with this adverse heavy metal ions impact by up-regulation of the MPST expression in the brain (lead, mercury), kidneys (lead, mercury) and skeletal muscle (lead, mercury, cadmium) (Table 5, Figure S3 Supporting information). The changes of the TST activity in various tissues were more complex. In the heart (lead), liver (mercury) and testes (cadmium), the TST activity was increased, while in the same time in the heart (cadmium) and skeletal muscle (lead, cadmium), it was decreased. The expression of TST gene was up-regulated in the brain (lead, mercury), kidney (lead, cadmium) and skeletal muscle (lead, mercury, cadmium) and down-regulated in the testes (mercury) (Table 5, Figure S2 Supporting information). Mercury ions also down-regulated the expression of MPST and CTH genes (Table 5, Figures S3 and S4 Supporting information). Based on the obtained results we can state that sulfurtransferases activity is regulated at the transcription level (Table 5). This regulation allowed the cells to increase the synthesis of needed proteins or to inhibit the synthesis when harmful intermediates were created in excess. Organisms adapt to environmental changes precisely by changing the expression of their genes and activity of their proteins.

The mechanism of lead, mercury and cadmium toxicity is complex and correlated with oxidative damages [54] of the cell structures, including DNA and cell membranes. Our results demonstrated different sensitivities and transcriptional responses of the investigated tissues to each of the heavy metals, as presented in Table 5. The antioxidant and stress-responsive gene expression was modulated in response to environmentally-relevant concentrations of heavy metals. The CTH gene up-regulation was confirmed in the liver (lead, mercury, cadmium) and skeletal muscle (mercury), MPST in the brain (lead, mercury), kidney (lead, cadmium), skeletal muscle (lead, mercury, cadmium) and TST in the brain (lead), kidney (lead, mercury, cadmium) (Table 4, Figures S2–S4 Supporting information). Thus, they can be regarded as heavy metal stress-responsive enzymes. Lead, mercury and cadmium toxicity have been demonstrated to affect the GSH level, biosynthesis of Cys and an altered [GSH]/[GSSG] balance (Table 6). Their toxicity also affects the levels of sulfane sulfur-containing compounds (Table 4, Figure S1 Supporting information), which in case of the enhanced ROS generation may show their antioxidative properties [11]. Jia et al. [55] suggested that the H_2_S and Cys cycle system is a key regulator of the response to cadmium-stress in plants that acts to induce and maintain levels of bioactive molecules (H_2_S, Cys, GSH and MTs) that improve plant resistance to cadmium-stress. Our results confirmed some of the results by Jia et al. [55], namely, that the CTH gene expression up-regulation results in the elevated Cys level in the liver (Table 4 and Table 5), Similarly, the MPST gene expression up-regulation results in the elevated Cys and GSH levels (Table 4 and Table 5). The present report is the first to widely describe the role of the sulfane sulfur/H_2_S generating enzymes and the Cys/glutathione system in Pb^2+^, Hg^2+^ and Cd^2+^ stress, in various frog tissues, and to explore the mechanisms mediating heavy metal related stress. 

## Figures and Tables

**Table 1:** Sequences of oligonucleotide primers used for detection target genes by reverse transcription polymerase chain reaction (RT-PCR).

Target Gene	No. in NCBI Database	Primers* (5’→3’) (Sense and Antisense)	Location in Gene	Product Size [bp]
**CTH**	NM_203706.1	ACCCAGGACTGCCGTCTCACC GCGCTGCTCTGTCCTCTTCTGG	888–1115	228
**MPST**	NM_001005646.1	ATGCAGCGACCGCACTTCCC CTTCTCGGGGCTCCGGCTCT	217–623	407
**TST**	NM_001103038.1	GTGACTGCGGAGGTGCCCAA TCTGGTTGGGCGCGGTGAAA	421–854	434
**GAPDH**	NM_001004949.2	GGGCGATGCTGGTGCAGTGT TCTGACCGGCACCTCTGCCA	281–633	353
**Cytoplasmic SOD**	NM_001016252.1	AACGGCTGTATCAGCGCGGG ATCACGCCACAAGCCAGGCG	176–456	281
**Mitochondrial SOD**	NM_001005694.1	TCTGCAGCCCCACATCAGTGC TCCCCACCCTGATCCCTGGAC	159–492	334
**GPx**	NM_001015740.2	ACAAGGGCAGAGTGCTTCTC CCACTGTTCTCCTGCTGTCC	138–297	160
**Cat**	NM_001011417.1	AGGTGGAGCAGATTGCCTTC CGGCATATGGCTGGGGTTAT	968–1191	224
**TrxR**	NM_001256471.1	CACCCCTTGGCACAAAATGG CCAAGTCCTGCCAAAAAGCC	661–1159	499

* All of the primers were synthesized by the DNA Sequencing and Synthesis Service—IBB PAN in Warsaw, Poland.

**Table 2:** PCR conditions for particular target genes.

Gene	Initial Denaturation		Denaturation		Amplification		cDNA Synthesis		Final Incubation		Amount of Cycles
**CTH**						60 s				10 min	30
**MPST**											28
**GAPDH**											
**Mitochondrial SOD**					58 °C					8 min	
**Cytoplasmic SOD**	94 °C	5 min	94 °C	30 s		30 s	72 °C	2 min	72 °C		26
**TST**											27
**GPx**					57 °C					5 min	29
**Cat**											
**TrxR**					62 °C			3 min			32

**Table 3:** Heavy metal content in *Pelophylax ridibundus* after 10-day exposition to Pb(NO_3_)_2_ (28 mg/L), HgCl_2_ (1.353 mg/L) and CdCl_2_∙2.5H_2_O (40 mg/L). Values represent the arithmetic mean ± SD of three–five animals, with each determination consisting of 3–5 assays.

Group	Pb(NO_3_)_2_	HgCl_2_	CdCl_2_· 2.5H_2_O
	[μg/kg of Dry Tissue]	[mg/kg of Dry Tissue]	
**BRAIN**			
**Control**	< LOQ	0.3^a^	2.5 ± 1.4
**Examined**	97.2^a^	16.8 ± 9.4	2.9 ± 1.6
**HEART**			
**Control**	<LOQ	<LOQ	2.2 ± 0.0
**Examined**	43.3^a^	166.0 ± 35.5	20.0 ± 7.5
**KIDNEY**			
**Control**	249.9 ± 196.7	0.8^a^	14.6 ± 3.2
**Examined**	4220.1 ± 852.0	2256.0 ± 906.0	453.9 ± 64.5
**LIVER**			
**Control**	48.7 ± 24.8	1.5 ± 1.0	49.0 ± 4.4
**Examined**	880.4 ± 217.0	559.1 ± 293.0	141.3 ± 67.8
**SKELETAL MUSCLE**			
**Control**	< LOQ	< LOQ	0.6 ± 0.0
**Examined**	7.7 ± 27.7	48.8 ± 9.7	2.1 ± 0.4
**TESTES**			
**Control**	lack of males in the group		
**Examined**	39.5 ± 18.7	118.6 ± 59.3	11.6^a^

^a^—standard deviation was not counted because of a low number of quantitative results. The lead level was determined by ICP-MS; mercury level by Hg-AAS and cadmium level using the F-AAS method. The limit of quantification (LOQ) for the lead—0.1 [µg/L]; mercury—1 [µg/L] and cadmium—10 [µg/L].

**Table 4:** Sulfane sulfur level and sulfurtransferases activity in *Pelophylax ridibundus* after 10-day exposition to Pb(NO_3_)_2_ (28 mg/L), CdCl_2_∙2.5H_2_O (40 mg/L) and HgCl_2_ (1.353 mg/L). Values represent the arithmetic mean ± SD of four–six animals, with each determination consisting of five–42 assays. * *p* < 0.05; ** *p* < 0.01; *** *p* < 0.001 (Student t-test).

Groups	Sulfane Sulfur	TST	MPST	CTH
	[nmol/mg Protein]	[nmol/mg Protein∙min]		
**BRAIN**				
**Control** ^**1**^	148.3 ± 36.7	334.0 ± 39.6	163.2 ± 20.2	0.09 ± 0.02
**Pb(NO**_**3**_ **)**_**2**_	224.1 ± 25.0***	364.0 ± 53.0	154.8 ± 15.6	0.30 ± 0.10*
**CdCl**_**2**_ **∙2.5H**_**2**_ **O**	203.6 ± 21.3***	352.2 ± 43.7	153.5 ± 8.7	0.17 ± 0.05*
**Control** ^**2**^	190.0 ± 28.7	400.0 ± 45.5	156.3 ± 5.6	0.12 ± 0.04
**HgCl**_**2**_	222.8 ± 18.0***	383.0 ± 50.0	61.1 ± 22.8***	0.51 ± 0.13***
**HEART**				
**Control** ^**1**^	136.6 ± 21.1	269.3 ± 21.4	490.5 ± 43.4	0.29 ± 0.13
**Pb(NO**_**3**_ **)**_**2**_	148.7 ± 9.9	599.7 ± 186.5***	387.9 ± 17.1***	0.29 ± 0.10
**CdCl**_**2**_ **∙2.5H**_**2**_ **O**	138.2 ± 24.8	234.6 ± 26.8***	425.2 ± 29.7***	0.21 ± 0.03
**Control** ^**2**^	109.7 ± 7.2	211.7 ± 40.9	292.7 ± 149.5	0.44 ± 0.31
**HgCl**_**2**_	101.0 ± 22.4	198.6 ± 36.9	158.6 ± 48.5***	0.18 ± 0.04*
**KIDNEY**				
**Control** ^**1**^	164.0 ± 22.9	2961.3 ± 683.6	722.4 ± 69.6	1.48 ± 0.28
**Pb(NO**_**3**_ **)**_**2**_	218.5± 16.5***	2810.9 ± 251.0	660.9 ± 35.1**	1.60 ± 0.24
**CdCl**_**2**_ **∙2.5H**_**2**_ **O**	179.6 ± 22.4*	2973.9 ± 645.7	604.3 ± 51.7***	1.23 ± 0.25*
**Control** ^**2**^	171.0 ± 26.1	3106.2 ± 348.4	745.2 ± 65.8	1.96 ± 0.49
**HgCl**_**2**_	156.8 ± 26.1*	3252.8 ± 365.0	695.6 ± 118.4	1.79 ± 0.35
**LIVER**				
**Control** ^**1**^	191.6 ± 26.9	1299.7 ± 306.0	619.6 ± 114.8	0.58 ± 0.12
**Pb(NO**_**3**_ **)**_**2**_	175.3 ± 24.7*	1449.9 ± 324.2	579.3 ± 116.7	0.71 ± 0.17*
**CdCl**_**2**_ **∙2.5H**_**2**_ **O**	166.3 ± 17.2***	1407.1 ± 321.2	510.6 ± 43.4***	0.52 ± 0.16
**Control** ^**2**^	171.3 ± 15.1	1080.8 ± 263.2	156.4 ± 38.9	0.45± 0.11
**HgCl**_**2**_	162.3 ± 18.3*	1418.9 ± 155.2***	161.2 ± 39.2	0.59 ± 0.15***
**SKELETAL MUSCLE**				
**Control** ^**1**^	75.7 ± 7.9	116.5 ± 29.7	99.4 ± 10.1	0.18 ± 0.13
**Pb(NO**_**3**_ **)**_**2**_	712.0 ± 18.5	86.6 ± 11.3***	90.9 ± 13.0*	0.28 ± 0.13
**CdCl**_**2**_ **∙2.5H**_**2**_ **O**	77.6± 12.1	94.1 ± 11.1***	88.7 ± 7.7***	0.11 ± 0.02
**Control** ^**2**^	50.8 ± 9.4	147.3 ± 35.7	31.8 ± 9.7	0.20 ± 0.11
**HgCl**_**2**_	61.1 ± 10.2***	170.9 ± 83.1	32.5 ± 8.5	0.36 ± 0.13**
**TESTES**				
**Control** ^**1**^	188.3 ± 23.9	51.8 ± 3.5	233.4 ± 35.1	0.19 ± 0.11
**Pb(NO**_**3**_ **)**_**2**_	196.7 ± 45.6	50.1 ± 7.1	208.9 ± 14.8*	0.09 ± 0.01
**CdCl**_**2**_ **∙2.5H**_**2**_ **O**	166.6 ± 28.2*	64.8 ± 16.8**	222.7 ± 8.0	0.09 ± 0.02
**Control** ^**2**^	180.0 ± 22.2	119.2 ± 3.8	208.2 ± 9.3	0.08 ± 0.00
**HgCl**_**2**_	206.3 ± 28.1*	129.1 ± 35.1	222.4 ± 55.8	0.19 ± 0.07**

^1^ Control group dedicated for experiment with lead and cadmium ions. ^2^ Control group dedicated for experiment with mercury ions.

**Table 5:** Expression of selected antioxidant genes in various tissues of *Xenopus tropicalis* after 10 days of exposition to heavy metal ions. The results are representative and obtained from four–five animals, with each determination consisting of two to nine tests.

	BRAIN	HEART	KIDNEY	LIVER	SKELETAL MUSCLE	TESTES
	Ctr Pb^2+^ Hg^2+^ Cd^2+^	Ctr Pb^2+^ Hg^2+^ Cd^2+^	Ctr Pb^2+^ Hg^2+^ Cd^2+^	Ctr Pb^2+^ Hg^2+^ Cd^2+^	Ctr Pb^2+^ Hg^2+^ Cd^2+^	Ctr Pb^2+^ Hg^2+^ Cd^2+^
**CTH**	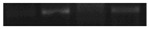	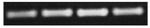	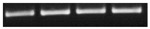	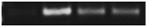	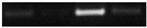	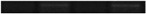
**MPST**	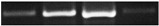	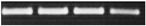	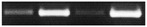	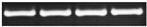	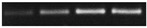	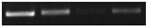
**TST**	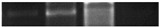	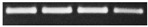	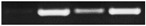	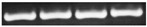	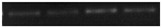	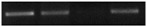
**Cytoplasmic SOD**	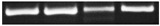	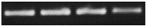	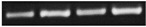	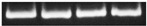	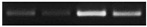	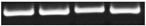
**Mitochondrial SOD**	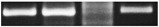	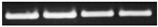	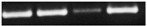	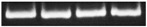	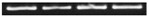	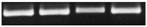
**GPx**	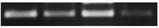	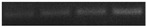	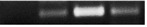	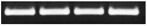	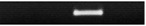	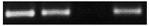
**Cat**	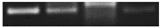	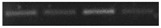	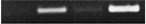	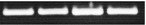	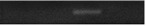	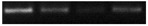
**TrxR**	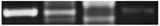	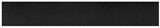	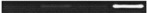	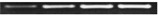	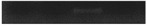	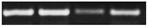
**GAPDH**	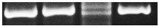	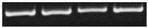	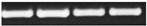	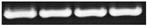	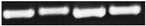	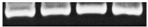

* Ctr—control group. Summarizing the results of the effect of the different metals on the selected antioxidant genes expression in various tissues of *Xenopus tropicalis* after 10 days of exposition are in the Supporting Information file—Table S1.

**Table 6:** Changes in the level of the oxidized and reduced form of glutathione, cysteine and cystine, total glutathione and total cysteine and the ratio between the reduced form of glutathione/cystine to the oxidized form in particular tissues of *Pelophylax ridibundus* (experiments with lead and cadmium ions) and *Xenopus tropicalis* (experiment with mercury ions) after 10 days of exposition to heavy metal ions. The data presented are the arithmetic means± SD of four–six animals (*Pelophylax ridibundus*) or three–four animals (*Xenopus tropicalis*), with each determination consisting of one–nine assays.

Group	GSH	GSSG	Total Glutathione (2GSSG+GSH)	GSH/GSSG	Cys	CSSC	Total Cysteine (2CSSC+Cys)	Cys/CSSC
	nmol/mg Protein				nmol/mg Protein			
**BRAIN**								
**Control**	20.3 ± 1.3	31.8 ± 2.0	83.8 ± 5.3	0.6 ± 0.0	3.7 ± 1.0	0.8^a^	6.3^a^	6.0^a^
**Pb(NO**_**3**_ **)**_**2**_	17.8 ± 3.8	27.1 ± 2.5	73.5 ± 12.6	0.6 ± 0.1	2.4 ± 0.5	0.8 ± 0.04	4.8 ± 1.1	3.0 ± 0.9
**Control**	16.8^a^	33.7^a^	84.1^a^	0.5^a^	3.7^a^	< LOD	NA	NA
**HgCl**_**2**_	5.9 ± 0.5	25.3 ± 1.3	56.4 ± 3.1	0.2 ± 0.0	1.4 ± 0.1	< LOD	NA	NA
**Control**	16.8 ± 4.3	30.4 ± 1.6	77.4 ± 6.3	0.6 ± 0.1	3.4 ± 0.2	< LOD	NA	NA
**CdCl**_**2**_ **∙2.5H**_**2**_ **O**	14.8 ± 1.7	28.5 ± 1.4	71.5 ± 4.7	0.5 ± 0.1	3.5 ± 0.4	< LOD	NA	NA
**HEART**								
**Control**	10.7 ± 2.3	3.5 ± 0.7	17.6 ± 3.7	3.1 ± 0.2	< LOD	<LOQ	NA	NA
**Pb(NO**_**3**_ **)**_**2**_	7.3 ± 1.4	2.2 ± 0.4	11.5 ± 2.2	3.5 ± 0.7	< LOD	<LOQ	NA	NA
**Control**	9.2^a^	4.0^a^	17.2^a^	2.3^a^	5.0^a^	<LOQ	NA	NA
**HgCl**_**2**_	8.4 ± 0.3	3.5 ± 0.4	16.4 ± 0.7	2.7 ± 0.3	5.3 ± 0.1	<LOQ	NA	NA
**Control**	4.8 ± 0.5	1.5 ± 0.4	7.8 ± 1.3	3.4 ± 0.7	<LOD	<LOD	NA	NA
**CdCl**_**2**_ **∙2.5H**_**2**_ **O**	7.0 ± 1.5	3.1 ± 0.2	13.2 ± 1.7	2.2 ± 0.4	< LOQ	<LOD	NA	NA
**KIDNEY**								
**Control**	10.6 ± 3.0	2.0 ± 0.3	14.5 ± 3.6	5.3 ± 0.7	8.1 ± 2.3	< LOQ	NA	NA
**Pb(NO**_**3**_ **)**_**2**_	7.6 ± 2.4	1.9 ± 0.2	11.3 ± 2.7	4.1 ± 0.9	10.1 ± 1.7	< LOQ	NA	NA
**Control**	9.1 ± 0.2	2.5 ± 0.2	14.1 ± 0.6	3.6 ± 0.2	4.9 ± 0.0	< LOQ	NA	NA
**HgCl**_**2**_	13.4 ± 1.9	2.6 ± 0.3	18.7 ± 2.2	5.2 ± 0.7	5.1 ± 0.1	< LOQ	NA	NA
**Control**	2.6 ± 0.3	1.4 ± 0.2	5.3 ± 0.7	1.9 ± 0.1	4.2 ± 0.5	< LOQ	NA	NA
**CdCl**_**2**_ **∙2.5H**_**2**_ **O**	12.7 ± 2.1	2.4 ± 0.3	17.4 ± 2.5	5.4 ± 0.7	5.5 ± 1.4	< LOQ	NA	NA
**LIVER**								
**Control**	11.6 ± 4.2	1.9 ± 0.3	15.4 ± 4.7	6.5 ± 0.6	1.2 ± 0.1	1.0 ± 0,1	3.5 ± 1.7	1.2 ± 0.6
**Pb(NO**_**3**_ **)**_**2**_	12.2 ± 3.2	2.0 ± 0.3	16.0 ± 3.5	6.3 ± 1.3	2.6 ± 0.6	2.5 ± 0.9	7.5 ± 2.0	1.1 ± 0.4
**Control**	3.3 ± 1.0	2.1 ± 1.1	6.3 ± 0.8	2.3 ± 0.8	1.4 ± 0.4	0.4 ± 0.0	2.1 ± 0.3	3.7 ± 1.3
**HgCl**_**2**_	14.0 ± 1.4	2.2 ± 0.6	18.4 ± 0.3	6.7 ± 2.1	1.2 ± 0.1	0.4 ± 0.0	1.9 ± 0.3	2.8 ± 0.2
**Control**	10.3 ± 2.4	2.3 ± 0.6	14.8 ± 2.1	4.9 ± 2.0	1.5 ± 0.3	1.1 ± 0.4	3.7 ± 1.0	1.4 ± 0.3
**CdCl**_**2**_ **∙2.5H**_**2**_ **O**	14.5 ± 2.8	1.6 ± 0.2	17.9 ± 2.9	8.6 ± 1.5	3.0 ± 0.4	0.9 ± 0.1	4.8 ± 0.5	3.1 ± 0.5
**SKELETAL MUSCLE**								
**Control**	2.8 ± 0.4	< LOQ	NA	NA	3.5^a^	< LOQ	NA	NA
**Pb(NO**_**3**_ **)**_**2**_	2.8 ± 0.4	0.1 ± 0.1	3.0 ± 0.5	36.2 ± 20.7	<LOD	< LOQ	NA	NA
**Control**	3.7 ± 0.8	0.6 ± 0.0	5.1 ± 1.0	6.7 ± 1.2	3.6 ± 0.2	< LOQ	NA	NA
**HgCl**_**2**_	3.6 ± 0.6	0.3 ± 0.1	3.5 ± 0.5	12.7 ± 3.7	3.1^a^	< LOQ	NA	NA
**Control**	2.3 ± 0.1	0.5 ± 0.1	3.5 ± 0.2	4.6 ± 1.3	3.7^a^	< LOQ	NA	NA
**CdCl**_**2**_ **∙2.5H**_**2**_ **O**	2.3 ± 0.1	< LOQ	NA	NA	<LOD	<LOQ	NA	NA
**TESTES**								
**Control**	lack of males in the group							
**Pb(NO**_**3**_ **)**_**2**_	18.2 ± 1.7	2.4 ± 0.1	22.9 ± 1.5	7.7 ± 1.0	2.0 ± 0.0	0.8 ± 0.0	3.5 ± 0.1	2.6 ± 0.1
**Control**	28.6^a^	3.9^a^	36.4^a^	7.3^a^	3.0^a^	<LOD	NA	NA
**HgCl**_**2**_	21.3 ± 3.3	3.2 ± 0.3	27.7 ± 2.8	6.7 ± 1.6	2.5 ± 0.1	1.3 ± 0.0	5.0 ± 0.0	1.9 ± 0.1
**Control**	14.0 ± 6.1	2.9 ± 0.3	19.7 ± 6.7	4.7 ± 1.7	1.4 ± 0.1	0.8 ± 0.0	3.0 ± 0.1	1.8 ± 0.1
**CdCl**_**2**_ **∙2.5H**_**2**_ **O**	60.9 ± 9.2	4.9 ± 0.5	70.6 ± 10.1	12.5± 0.7	3.0 ± 0.5	<LOD	NA	NA

^a^—standard deviation was not counted because of a low number of quantitative results; <LOD, lower than the limit of detection of the method; <LOQ, lower than the limit of quantification of the method; NA, not applied. The limit of detection for glutathione (GSH) in the RP-HPLC method is equal to 0.01 [nM·mL^-1^] and for oxidized form of glutathione (GSSG)—0.1 [nM·mL^-1^]. The limit of quantification for GSH 0.1 [nM·mL^-1^] and GSSG: 1 [nM·mL^-1^] [35]. The limit of detection for cysteine (Cys) was defined in the RP-HPLC method and is equal to 0.01 [nM·mL^-1^] and for cystine (CSSC)—0.1 [nM·mL^-1^]. The limit of quantification for Cys: 0.1 [nM·mL^-1^] and CSSC—1 [nM·mL^-1^] [35].

**Table 7:** Changes in the concentration of malondialdehyde in particular tissues of *Pelophylax ridibundus* after 10 days of exposition to heavy metal ions. The data presented are the arithmetic mean ± SD of three–four animals, with each determination consisting of three assays.

	MDA [nM/g of Wet Tissue]			
	Control	Pb(NO_3_)_2_	HgCl_2_	CdCl_2_∙2.5H_2_O
**BRAIN**	73 ± 7	34 ± 2	47 ± 8	78 ± 22
**HEART**	61 ± 9	47 ± 8	53 ± 14	46 ± 9
**KIDNEY**	114 ± 15	105 ± 5	116 ± 25	94 ± 17
**LIVER**	226 ± 27	261 ± 5	278 ± 53	134 ± 26
**SKELETAL MUSCLE**	5^a^	6 ± 3	3 ± 2	3 ± 2
**TESTES**	65^a^	90^a^	70 ± 56	lack of male in the group

^a^—standard deviation was not counted because of a low number of results.

## Supplementary Materials

The following are available online at https://www.mdpi.com/2218-273X/10/4/574/s1, Figure S1: Sulfane sulfur level (*Pelophylax ridibundus*) and the level of glutathione and cysteine (*Pelophylax ridibundus, Xenopus tropicalis*) and MDA level (*Pelophylax ridibundus*) in the frogs’ tissues after 10 days exposition to heavy metal compounds. The activity values for the control and experimental groups are in Table 4. Figure S2: Rhodanase activity (*Pelophylax ridibundus*) and expression (*Xenopus tropicalis*) in different frogs’ tissues after 10 days exposition to heavy metal compounds. The activity values for the control and experimental groups are in Table 4. Figure S3: 3-mercaptopyruvate sulfurtransferase activity (*Pelophylax ridibundus*) and expression (*Xenopus tropicalis*) in different frogs’ tissues after 10 days exposition to heavy metal compounds. The activity values for the control and experimental groups are in Table 4. Figure S4: Cystathionine γ-lyase activity (*Pelophylax ridibundus*) and expression (*Xenopus tropicalis*) in different frogs’ tissues after 10 days exposition to heavy metal compounds. The activity values for the control and experimental groups are in Table 4. Table S1: Summarizing the effect of the different metals on the selected antioxidant genes expression in various tissues of *Xenopus tropicalis* after 10 days of exposition.
