# Supplementary material for: Multidirectional Changes in Parameters Related to Sulfur Metabolism in Frog Tissues Exposed to Heavy Metal-Related Stress
Source: Biomolecules. 2020 Apr 9;10(4):574. doi: 10.3390/biom10040574 (PMC7226484; doi:10.3390/biom10040574)
Supplement: Supplementary file 1 [file biomolecules-10-00574-s001.pdf]

Supporting information

Multidirectional changes in parameters related to sulfur metabolism in frog tissues exposed to heavy metal-related stress

Marta Kaczor-Kamińska, Piotr Sura and Maria Wróbel*

1. Results section

Results from the manuscript presented in a different way.

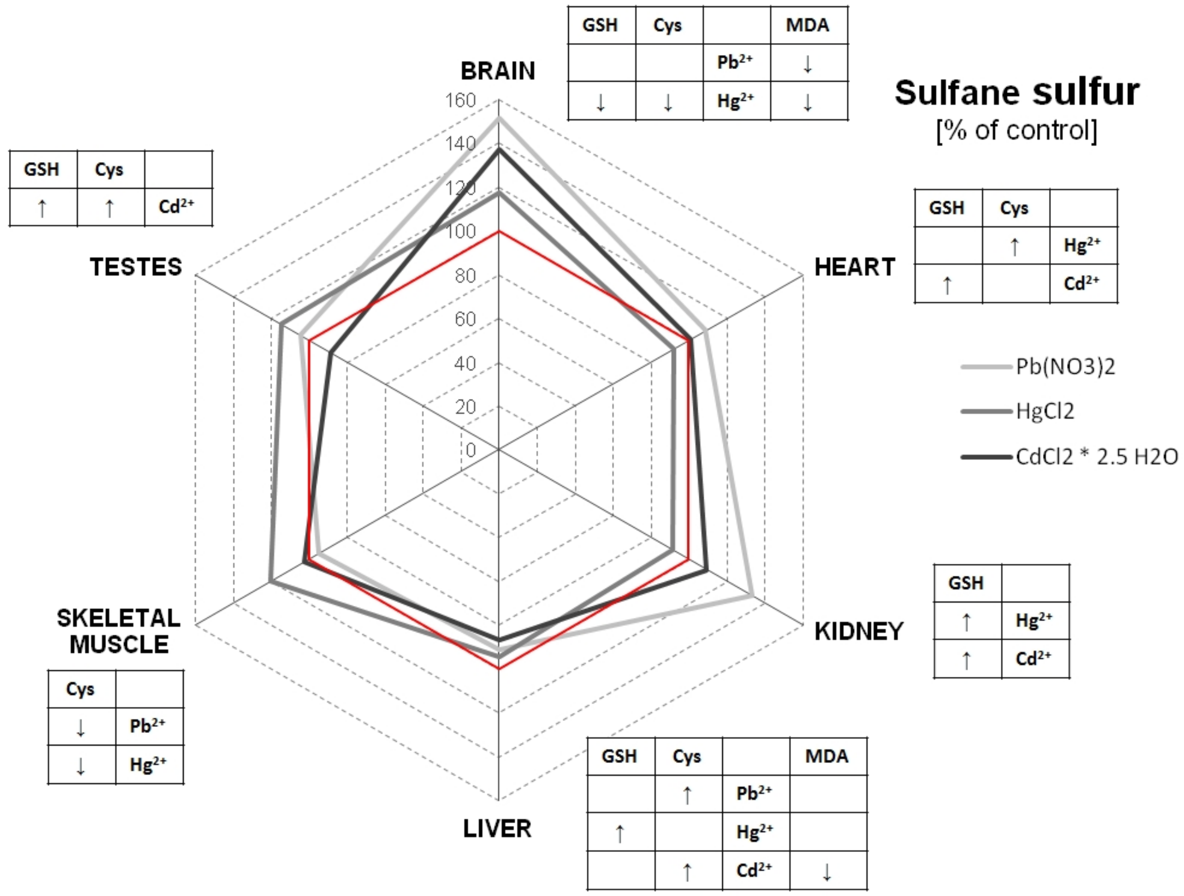

Figure S1. Sulfane sulfur level (*Pelophylax ridibundus*) and the level of glutathione and cysteine (Pelophylax ridibundus, Xenopus tropicalis) and MDA level (Pelophylax ridibundus) in the frogs' tissues after 10 days exposition to heavy metal compounds. The activity values for the control and experimental groups are in Table 4.

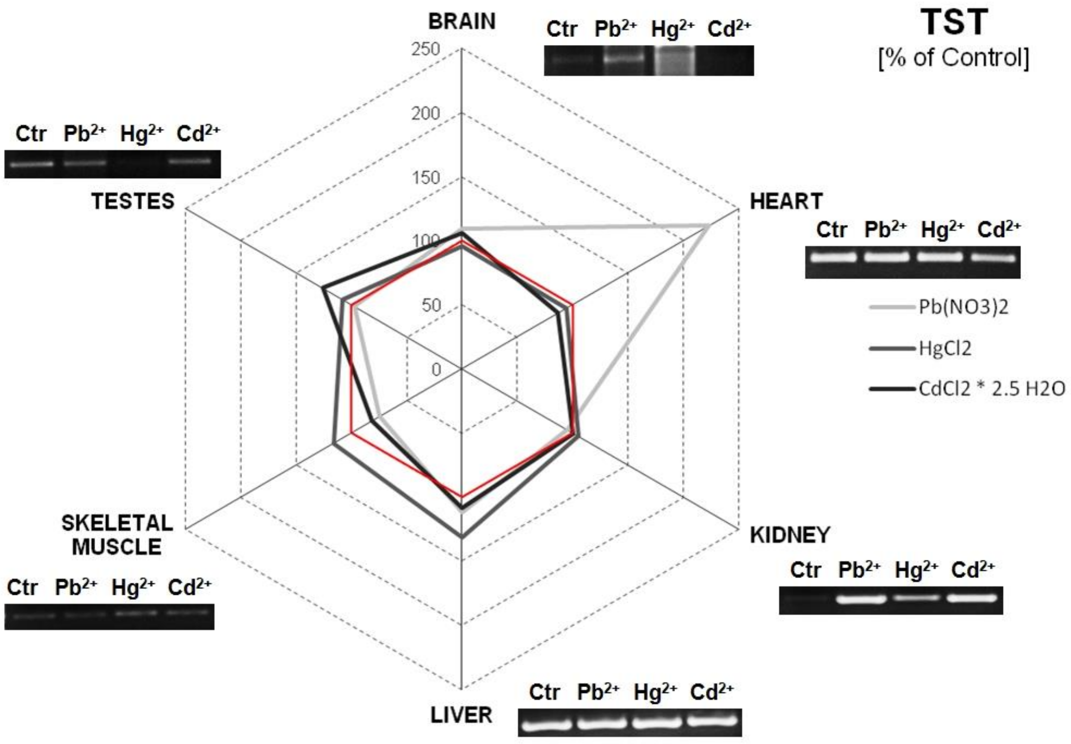

Figure S2. Rhodanase activity (*Pelophylax ridibundus*) and expression (*Xenopus tropicalis*) in different frogs' tissues after 10 days exposition to heavy metal compounds. The activity values for the control and experimental groups are in Table 4.

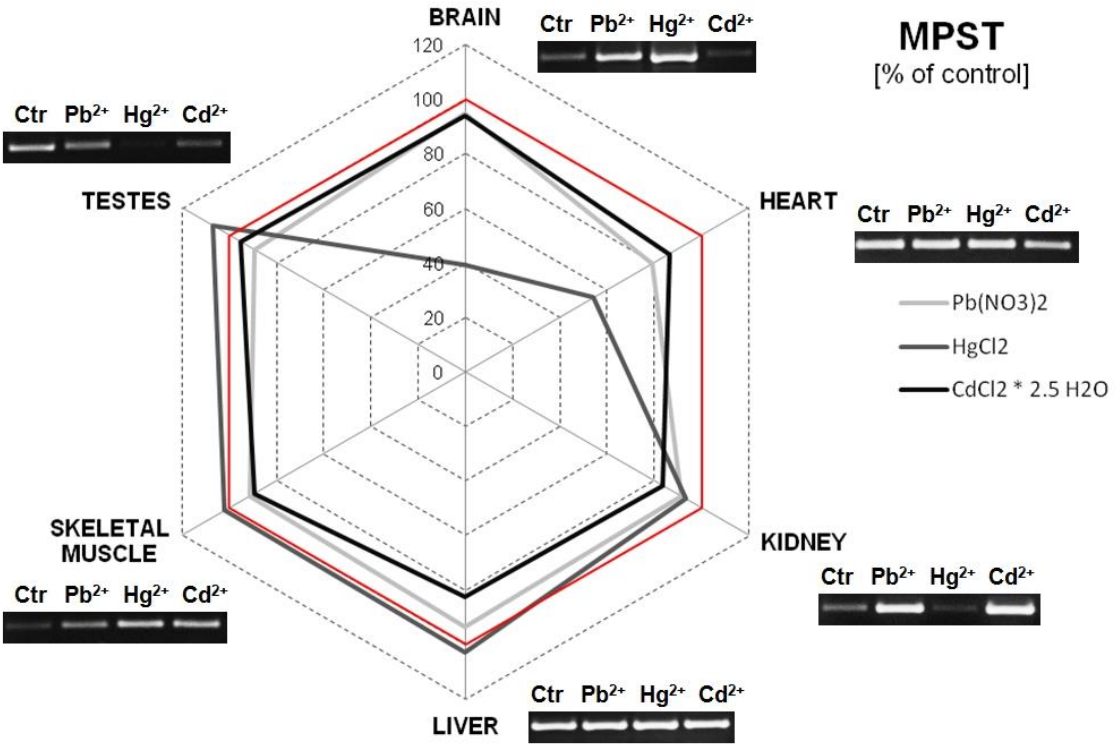

Figure S3. 3-mercaptopyruvate sulfurtransferase activity (*Pelophylax ridibundus*) and expression (*Xenopus tropicalis*) in different frogs' tissues after 10 days exposition to heavy metal compounds. The activity values for the control and experimental groups are in Table 4.

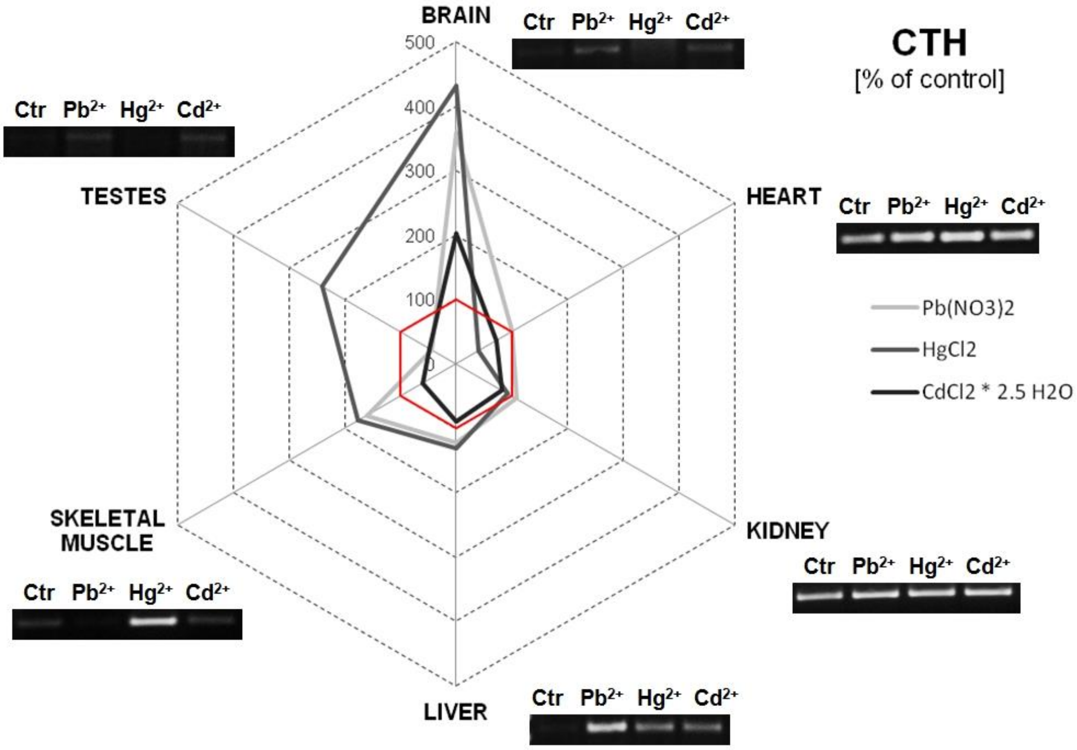

Figure S4. Cystathionine γ-lyase activity (*Pelophylax ridibundus*) and expression (*Xenopus tropicalis*) in different frogs' tissues after 10 days exposition to heavy metal compounds. The activity values for the control and experimental groups are in Table 4.

Table S1. Summarizing the effect of the different metals on the selected antioxidant genes expression in various tissues of *Xenopus tropicalis* after 10 days of exposition

	**BRAIN**				**HEART**				**KIDNEY**				**LIVER**				**SKELETAL MUSCLE**				**TESTES**			
	**Ctr**	**Pb^{2+}**	**Hg^{2+}**	**Cd^{2+}**	**Ctr**	**Pb^{2+}**	**Hg^{2+}**	**Cd^{2+}**	**Ctr**	**Pb^{2+}**	**Hg^{2+}**	**Cd^{2+}**	**Ctr**	**Pb^{2+}**	**Hg^{2+}**	**Cd^{2+}**	**Ctr**	**Pb^{2+}**	**Hg^{2+}**	**Cd^{2+}**	**Ctr**	**Pb^{2+}**	**Hg^{2+}**	**Cd^{2+}**
**CTH**	○	○	-	○	○	○	○	○	○	○	○	○	<->	+	+	+	○	○	+	○	<->	○	<->	○
**MPST**	<->	+	+	<->	○	○	○	-	○	+	○	+	○	○	○	○	○	+	+	+	○	○	-	○
**TST**	○	+	<->	<->	○	○	○	-	○	+	+	+	○	○	○	○	○	○	○	○	○	○	-	○
**Cytoplasmic SOD**	○	○	-	○	○	○	○	-	○	○	○	○	○	○	○	○	○	○	+	+	○	○	○	○
**Mitochondrial SOD**	○	○	<->	-	○	○	○	○	○	○	-	○	○	○	○	○	○	○	○	○	○	○	○	○
**GPx**	○	○	○	-	○	○	○	○	<->	+	+	+	○	○	○	○	<->	<->	+	<->	○	○	<->	○
**Cat**	○	○	<->	-	○	○	○	○	○	+	○	+	○	○	○	○	<->	<->	+	<->	○	○	-	○
**TrxR**	○	-	<->	-	<->	<->	<->	<->	○	○	○	+	○	+	+	+	<->	<->	+	<->	○	○	-	○
**GAPDH**	○	○	<->	○	○	○	○	○	○	○	○	○	○	○	○	○	○	○	○	○	○	○	○	○

band presence (○); up-regulation (+); down-regulation(-); no band (<->)
